# Carrier-Free Supramolecular Hydrogel Self-Assembled from Triterpenoid Saponins from Traditional Chinese Medicine: Preparation, Characterization, and Evaluation of Anti-Inflammatory Activity

**DOI:** 10.3390/gels12010052

**Published:** 2026-01-02

**Authors:** Qiongxue Huang, Mingzhen Liu, Tingting Ye, Dandan Mo, Haifeng Wu, Guoxu Ma, Xiaolei Zhou

**Affiliations:** 1 Key Laboratory of Bioactive Substances and Resource Utilization of Chinese Herbal Medicine, Ministry of Education, Institute of Medicinal Plant Development, Peking Union Medical College and Chinese Academy of Medical Sciences, Beijing 100193, China; 2 Guangxi Botanical Garden of Medicinal Plants, Nanning 530023, China

**Keywords:** α-hederin, self-assembly, traditional Chinese medicine, carrier-free

## Abstract

Inflammation is the body’s natural immune response to invasion by foreign pathogens and is closely linked to many diseases. Chronic inflammation, if not properly controlled, can pose serious health risks and even threaten life. Currently, the main anti-inflammatory drugs are classified into steroidal and non-steroidal anti-inflammatory drugs, but both have significant side effects that limit their clinical applications. α-Hederin, a pentacyclic triterpenoid saponin, is derived from various plants, including *Pulsatilla chinensis*, *Hedera helix*, and *Nigella sativa*. It has been reported that α-hederin can be used to treat both acute and chronic inflammatory diseases. However, it has poor water solubility and low bioavailability. This study shows that α-hederin can directly self-assemble into a hydrogel through hydrogen bonds and van der Waals forces, called He-Gel. The mechanical properties of He-Gel were further characterized using rheological and microrheological methods. Its self-assembly mechanism was comprehensively elucidated through a combination of spectroscopic analyses and computational chemistry. Furthermore, in vitro experiments showed that He-Gel exhibits lower cytotoxicity and more excellent anti-inflammatory activity compared to free α-hederin. In conclusion, this research provides a solution for the further development of α-hederin. Unlike conventional approaches that rely on polymers as drug carriers, this preparation method is both green and economical. More importantly, it highlights that direct self-assembly of natural small molecules represents a promising strategy for anti-inflammatory therapy.

## 1. Introduction

Inflammation, primarily categorized into chronic and acute inflammation according to its duration, is a pivotal component of defensive immune responses and typically occurs when foreign pathogens invade organisms. Acute inflammation typically resolves once the underlying cause improves, whereas chronic inflammation persists over time and can worsen pathological conditions [1]. Chronic inflammatory diseases have been identified as a leading cause of death worldwide, accounting for more than 50% of all fatalities related to inflammation-associated conditions [2]. Currently approved anti-inflammatory drugs in clinical practice are primarily classified into steroidal anti-inflammatory drugs (SAIDs) and nonsteroidal anti-inflammatory drugs (NSAIDs). However, their clinical utility is significantly constrained by notable adverse effects. For instance, glucocorticoids, a class of SAIDs, can induce iatrogenic Cushing’s syndrome, while NSAIDs are associated with a spectrum of gastrointestinal adverse reactions [3,4]. Consequently, there is an urgent demand to develop novel anti-inflammatory agents with enhanced safety and efficacy profiles.

Natural products remain an essential source for drug discovery, with phytochemical-derived agents such as vinblastine, camptothecin, paclitaxel, and artemisinin continuing to expand the spectrum of available therapies [5]. Among natural substances with notable anti-inflammatory effects, saponins, flavonoids, alkaloids, and polysaccharides are the most widely studied [6]. α-Hederin, a pentacyclic triterpenoid saponin derived from various medical plants such as *Pulsatilla chinensis*, *Hedera helix*, and *Nigella sativa*, exhibits diverse biological activities including anti-tumor [7], anti-inflammatory [8], and anti-fungal [9] properties. For example, Saadat et al. demonstrated that α-hederin protects against tracheal hyperreactivity and pulmonary inflammation in ovalbumin-sensitized guinea pigs [8], while Zeng et al. reported that it alleviates liver and lung inflammation in septic mice by modulating macrophage M1/M2 polarization and suppressing NF-κB activation [10]. Despite its promising pharmacological activity, the clinical application of α-hederin is hindered by its natural pentacyclic triterpene structure, which results in poor water solubility and low bioavailability. DMSO is commonly used to help dissolve substances for administration, but it has raised safety concerns such as liver and kidney damage, blood vessel narrowing, and neurotoxicity [11]. Sun et al. used polymeric micelles as an integrated platform for the targeted delivery of α-hederin to cancer cells, overcoming its drawback of poor water solubility [12]. Yet the synthesis method for the amphiphilic block copolymers used in this study is complex, and the polymers have a broad molecular weight distribution, which poses certain safety concerns. Wang et al. encapsulated α-hederin and oxaliplatin into recombinant high-density lipoprotein (rHDL)-modified liposomes, which effectively improved their solubility and bioavailability [13]. Similarly, the modification method used in this study is not only time-consuming and labor-intensive but also has the drawbacks of low drug loading capacity and the risk of premature off-target release in vivo, which can significantly reduce its therapeutic effectiveness.

Recently, supramolecular hydrogels formed by the self-assembly of natural small molecules derived from traditional Chinese medicines (TCMs) have gained considerable attention. These assemblies are typically stabilized through non-covalent interactions such as hydrogen bonding, π–π stacking, electrostatic forces, and van der Waals interactions. This emerging “self-delivery strategy” offers several advantages, including improved therapeutic efficacy, sustained release, and reduced toxicity. A typical example is that in 2019, Wang and his team found that rhein can self-assemble into a hydrogel through various non-covalent interactions under alkaline conditions. Rhein hydrogel has lower toxicity than free rhein and a more long-lasting therapeutic effect on neuroinflammation [14]. More recently, in 2025, Peng et al. demonstrated that Zingibroside R1 (ZR1), isolated from *Panax notoginseng*, could self-assemble into a hydrogel in water [15]. Notably, they employed cryo-electron microscopy to reveal, for the first time, the unique antifungal mechanism of ZR1 hydrogel compared with free ZR1. Inspired by these findings, we aim to explore whether α-hederin has the potential to self-assemble into a hydrogel, given its unique amphiphilic structure.

**Scheme 1:**
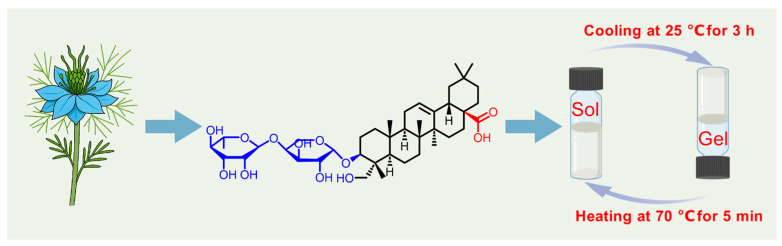
Diagram of chemical structure of α-hederin and sol–gel transition of He-Gel.

Herein, we report for the first time that α-hederin can directly self-assemble into a supramolecular hydrogel (He-Gel) through hydrogen bonding and van der Waals interactions (Scheme 1). The microscopic morphology of He-Gel was examined using electron microscopy, while its mechanical properties were characterized by rheology and microrheology. To further elucidate the assembly mechanism, we employed nuclear magnetic resonance (NMR), Fourier-transform infrared spectroscopy (FT-IR), X-ray diffraction (XRD), and zeta potential analysis, and these results were further supported and visualized by molecular dynamics (MD) simulations. We next evaluated the biological performance of He-Gel using RAW264.7 macrophages. Cytotoxicity was assessed after 24 h and 48 h, and inflammatory cytokine levels in the culture supernatant were measured at 24 h. The results demonstrated that He-Gel exhibited lower cytotoxicity and stronger anti-inflammatory activity compared with free α-hederin. Several plausible explanations are as follows: In the gel structure, the carboxyl groups are involved in hydrogen bond formation, which reduces the repulsive force between them and the cell membrane, thereby decreasing toxicity. Meanwhile, the microscale lamellar structure of the gel increases the contact area between the assembly and cells. Molecular dynamics (MD) simulations also reveal an increased number of hydrogen bonds in the gel. These factors collectively contribute to the reduced toxicity and enhanced therapeutic efficacy of the gel. Due to its simple preparation, environmentally friendly nature, and cost-effectiveness, this carrier-free gel holds significant promise for development as an anti-inflammatory drug.

## 2. Results and Discussion

### 2.1. Preparation and Characterization of He-Gel

**Figure 1:**
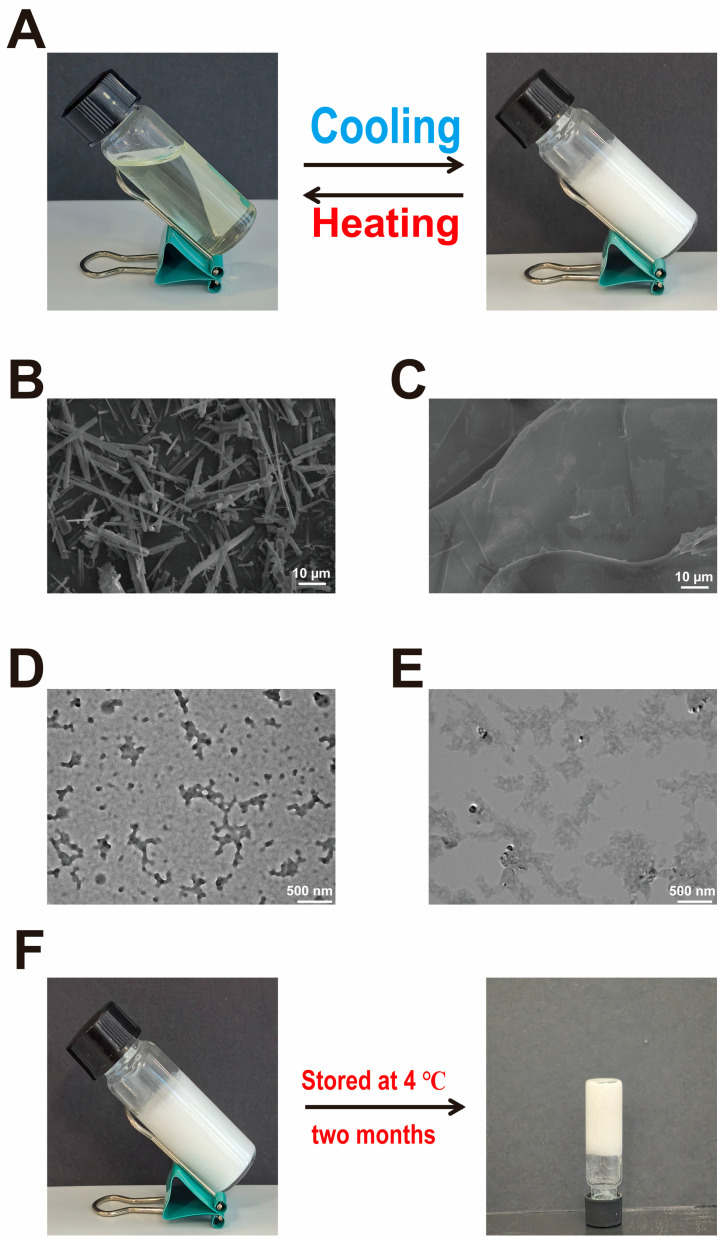
The morphology study of α-hederin and He-Gel. (**A**) The appearance of α-hederin dissolved in Na_2_CO_3_ solutions (sol: 70 °C; gel: 25 °C). (**B**) The SEM image of α-hederin (scale bar: 10 μm). (**C**) The SEM image of He-Gel (scale bar: 20 μm). (**D**) The TEM image of α-hederin (scale bar: 500 nm). (**E**) The TEM image of He-Gel (scale bar: 500 nm). (**F**) The stability detection of He-Gel.

He-Gel was prepared via a simple heating-cooling method (Figure 1B). Briefly, α-hederin powder was dissolved in Na_2_CO_3_ solution, with the temperature raised to 70 °C to obtain a transparent solution. This solution was then kept at room temperature for 3 h, during which α-hederin spontaneously self-assembled into an opaque white hydrogel. The formation of He-Gel was confirmed by the tube-inversion method. Subsequently, according to previous study [16], we performed control independent variable method to verify the critical gel concentration of α-hederin and Na_2_CO_3_. By varying the concentrations of α-hederin and Na_2_CO_3_, we identified the critical gelation concentration as 66.6 mM α-hederin in 0.1 M Na_2_CO_3_ solution. The microstructures of freeze-dried He-Gel and α-hederin monomer were then examined by electron microscopy. As shown in Figure 1C, scanning electron microscopy (SEM) revealed that freeze-dried He-Gel exhibited an intersecting lamellar structure, which was distinct from the cuboid-like microstructures observed in the α-hederin monomer (Figure 1D). Consistently, transmission electron microscopy (TEM) further confirmed these findings, showing sheet-like structures in He-Gel, in contrast to the spherical morphology of the α-hederin monomer (Figure 1E). We also conducted a preliminary assessment of the temporal stability of the He-Gel. Throughout the observation period, He-Gel retained its original conformation and hydration state, devoid of any visible degradation or discoloration. Such enduring stability suggests that the material possesses an excellent shelf-life profile, which is a critical attribute for its potential commercial or clinical applications (Figure 1F).

**Figure 2:**
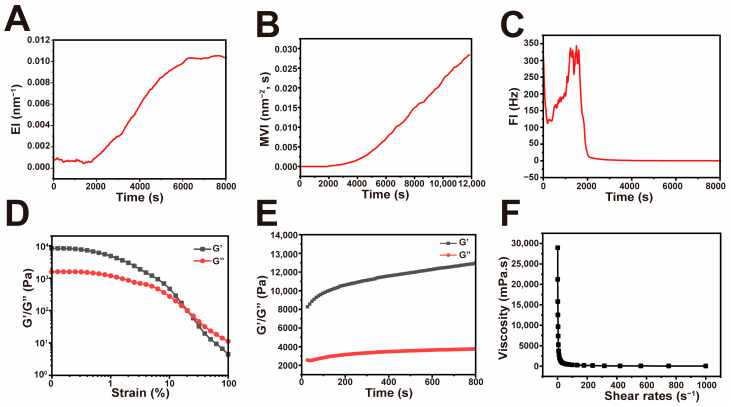
The micro-rheological and rheological results of He-Gel. (**A**) The curve of the elasticity index (EI) of He-Gel as a function of time at 25 °C in microrheological measurements. (**B**) The curve of the macroscopic viscosity index (MVI) of He-Gel as a function of time at 25 °C in microrheological measurements. (**C**) The curve of the fluidity index (FI) of He-Gel as a function of time at 25 °C in microrheological measurements. (**D**) Amplitude scanning results of He-Gel with the strain from 0.1% to 100% (T = 37 °C, frequency = 1 Hz). (**E**) Time sweep results of He-Gel at a single frequency when the strain was set at 1%, frequency at 1 Hz and temperature at 37 °C. (**F**) The shear-thinning property of He-Gel as the shear rate changes from 0.1 s^−1^ to 100 s^−1^ (T = 25 °C).

Micro-rheological and rheological tests were implemented to evaluate the gelation properties of He-Gel. Micro-rheological analysis can directly reflect the phase transition of the hydrogel in response to temperature changes through the elasticity index (EI), macroscopic viscosity index (MVI), and fluidity index (FI) [17]. As demonstrated in Figure 2A–C, EI and MVI gradually increased, while FI decreased within 2 h, intuitively presenting the forming process of He-Gel. The microrheological results indicate that He-Gel exhibits high viscosity and low fluidity, which provides significant advantages for drug delivery applications, especially for localized administration. Its limited mobility allows the gel to remain effectively at the target site without spreading to surrounding areas, thereby reducing the risk of damaging healthy tissues. Rheological testing, another standard to prove the formation of He-Gel, provides information on mechanical moduli such as storage modulus (G′) and loss modulus (G″), which are valuable for estimating application potential. To evaluate its potential applications in the biomedical field, we examined its rheological properties at 37 °C. The amplitude sweep results indicated that the critical strain point was 20%, meaning that He-Gel transformed into a sol state once the strain exceeded this value (Figure 2D). Next the strain was set at 1%, we examined the modulus changes with time. The result shown that G’ always positioned above G” which suggested He-Gel kept its gel state (Figure 2E). In addition, the shear-thinning property reflects the potential of gels for applications such as injection and spray. This property indicates that the gel can transition from a highly viscoelastic state at rest to a low-viscosity flowable state under mechanical stress. Such a response is highly desirable for administration modes that require transient fluidity—most notably injection or spray delivery. Under high shear (e.g., during passage through a needle or atomization), the gel can flow easily, while upon cessation of shear it rapidly recovers its structure, maintaining a stable depot-like form. As shown in Figure 2F, He-Gel exhibited a pronounced shear-thinning behavior, which is favorable for practical use. The favorable mechanical properties and shear-thinning behavior endow the gel with injectable and sprayable characteristics, which offer broad application prospects in the biomedical field, particularly for intratumoral injection and skin repair [18,19].

### 2.2. The Self-Assembly Mechanism of He-Gel

**Figure 3:**
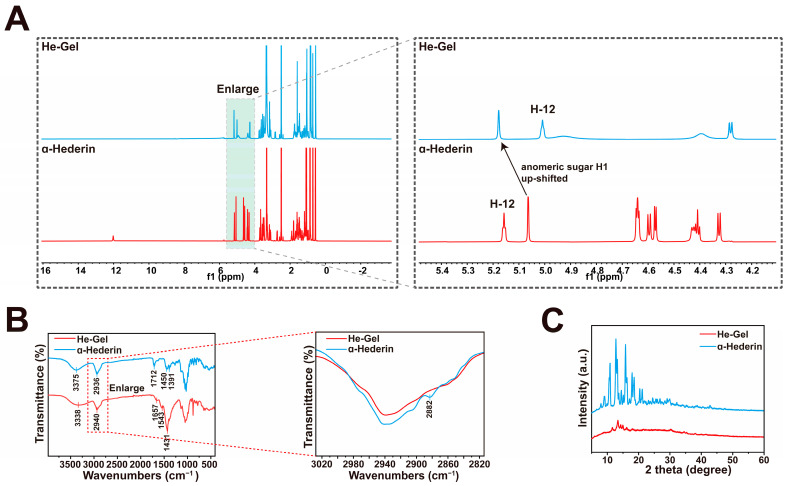
The self-assembly mechanism analysis of He-Gel. (**A**) The total and enlarged ^1^H NMR spectra of He-Gel and α-hederin at 25 °C. (**B**) The FT-IR spectra of He-Gel and α-hederin (left: the complete spectra; right: enlarged spectra from 3000 cm^−1^ to 2800 cm^−1^). (**C**) XRD spectra of He-Gel and α-hederin.

To further investigate the self-assembly mechanism of He-Gel, a series of spectral experiments were conducted. Proton nuclear magnetic resonance (^1^H NMR) is a powerful technique for identifying assembly forces in supramolecular systems, such as hydrogen bonding and π–π stacking interactions [20,21]. Thus, we first examined the self-assembly mechanism of He-Gel using ^1^H NMR at room temperature. As shown in Figure 3A,B, hydrogen signal peak at 5.00 (t, H-12) of He-Gel had a significant movement to high field in contrast to the peak of α-hederin which could be speculated the existence of π-π stacking interaction during the process of self-assembly. Additionally, compared with α-hederin, the anomeric sugar H1 exhibits a clear downfield shift, indicating an increase in chemical shift. This suggests the formation of hydrogen bonds maybe existing during the self-assembly process. The ^1^H NMR spectra in DMSO-d_6_ confirm the chemical stability of α-hederin during the gelation process. While DMSO disrupts the aqueous gel network, these spectral changes verify the generation of the amphiphilic building blocks required for the subsequent supramolecular assembly, which is further evidenced by FT-IR and rheological studies.

FT-IR spectroscopy can reveal changes in the characteristic peaks of functional groups, thereby elucidating the intermolecular interactions involved in the self-assembly process. As demonstrated in Figure 3B, the peak located at 3375 cm^−1^ of α-hederin could be assigned to the O-H stretching vibration, while the carbonyl (C=O) stretching vibration of the carboxylic acid group appears at 1712 cm^−1^. After self-assembly, the O-H stretching vibration peak red-shifted from 3375 cm^−1^ to 3338 cm^−1^, accompanied by reduced intensity and band broadening, clearly indicating that hydrogen bonding as one of the driving forces for self-assembly [22,23]. Furthermore, α-hederin exhibited a distinct asymmetric and symmetric stretching vibration of CH_2_ at 2936 and 2862 cm^−1^, while the symmetric stretching vibration peak in He-Gel disappeared along with the weakened asymmetric stretching peak [24]. The shift from 1712 cm^−1^ to 1657 cm^−1^ confirms the deprotonation of the carboxylic acid group to form the carboxylate anion. This ionization increases the amphiphilicity of the molecule, which is the driving force for the subsequent supramolecular self-assembly into the gel network. These results suggest that the triterpene skeleton of α-hederin underwent strong stacking interactions during the formation of He-Gel, likely stabilized by van der Waals forces.

X-ray diffraction (XRD) is widely employed to characterize the microstructure of both crystalline and amorphous materials, allowing the monitoring of polymorphic transitions during self-assembly processes. In our study, the majority of crystalline diffraction peaks disappeared upon gel formation of α-hederin, confirming the amorphous nature of the lyophilized gel powder.

It should be noted that the physicochemical characterizations reflect the properties of the formed supramolecular salt species compared to the neutral parent compound. Since the formation of the salt is intrinsically coupled with the self-assembly process at these concentrations, the observed spectral changes represent the cumulative effect of ionization and network formation.

### 2.3. Density-Functional Theory (DFT) Calculation of He-Gel

**Figure 4:**
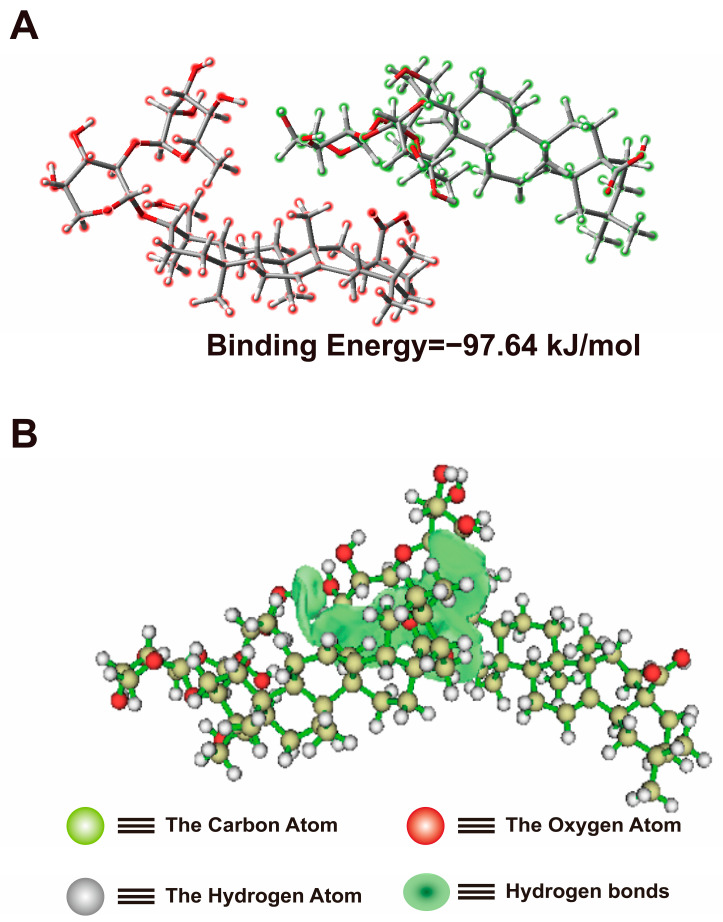
DFT calculation and IGMH analysis of He-Gel. (**A**) Image of two α-hederin molecules binding mode. (**B**) Image of IGMH analysis. The part of green indicate the hydrogen bonding interactions between α-hederin molecules.

Density-functional theory (DFT) enables us to further clarify the self-assembly mode of α-hederin molecules and determine the sites of possible action via different potentials distribution. As shown in Figure 4A, DFT calculations revealed that α-hederin molecules assembled in a stable anti-parallel stacking mode mediated by hydrogen bonding interactions. The optimized binding configuration obtained using Gaussian 16 showed a binding energy of –97.64 kJ/mol, confirming strong and stable intermolecular interactions. Visualization by the Independent Gradient Model based on Hirshfeld Partitioning (IGMH) further supported this result, as abundant green regions appeared between adjacent molecules, indicating extensive hydrogen bonding networks (Figure 4B). These results indicate that α-hederin molecules can form stable binding through hydrogen bonds and van der Waals forces.

### 2.4. Molecular Dynamics Simulation of He-Gel

**Figure 5:**
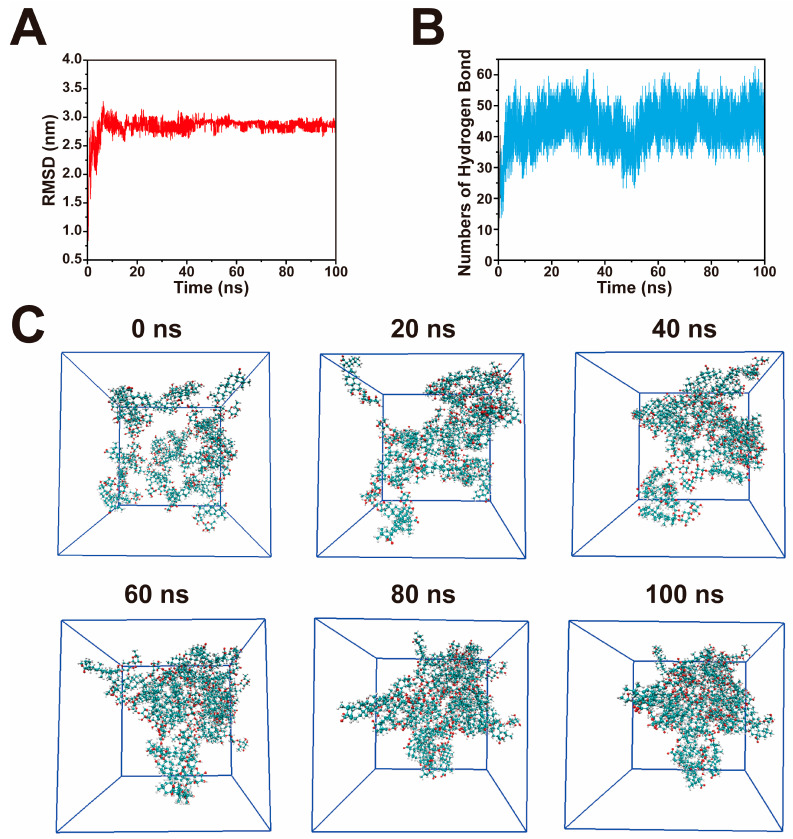
MD stimulation study of He-Gel. (**A**) The variation of RMSD over time during the simulated assembly process. (**B**) The variation of hydrogen bonding numbers over time during the simulated assembly process. (**C**) Graph of the variation of aggregation trajectories over time during the simulated assembly process.

**Figure 6:**
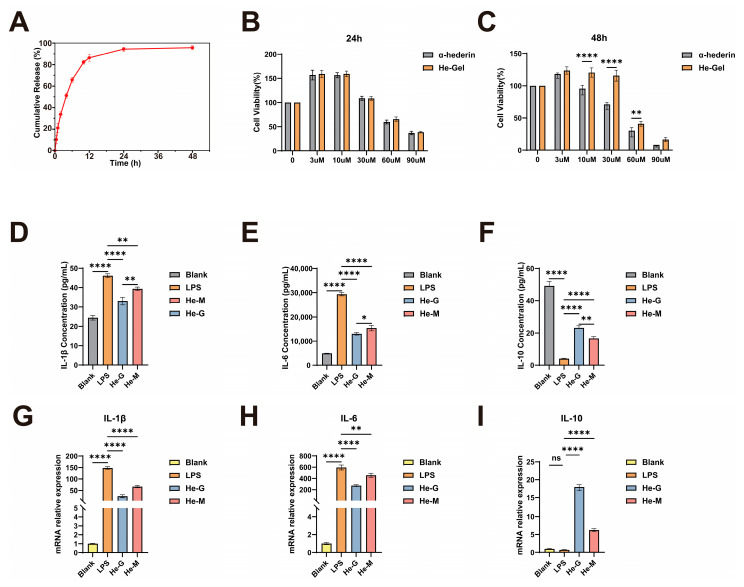
He-Gel performed sustainable release, lower cytotoxicity and better anti-inflammatory activity. (**A**) In vitro release curve of He-Gel and the concentration of He-Gel was 50 mg/mL. (**B**) Determination of the viability of RAW264.7 cells after 24 h treatment with α-hederin and He-Gel via CCK-8 assay. (**C**) Determination of the viability of RAW264.7 cells after 48 h treatment with α-hederin and He-Gel via CCK-8 assay. (**D**) The concentration of IL-1β in the supernatant of RAW264.7 cells at 24 h after different treatments was determined by ELISA. (**E**) The concentration of IL-6 in the supernatant of RAW264.7 cells at 24 h after different treatments was determined by ELISA. (**F**) The concentration of IL-10 in the supernatant of RAW264.7 cells at 24 h after different treatments was determined by ELISA. (**G**) mRNA expression level of IL-1β. (**H**) mRNA expression level of IL-6. (**I**) mRNA expression level of IL-10. The date is represented as the mean ± SD and one-way ANOVA and two-way ANOVA in GraphPad Prism 10.6.1 version was adopted to calculate the statistical significance (* *p* < 0.05, ** *p* < 0.01, **** *p* < 0.0001).

Numerous studies have shown that all-atom molecular dynamics simulation (MDS) can simulate the assembly process of molecules, which is conducive to the prediction of assemblies and the elaboration of their mechanisms. Hence, to better demonstrate the assembly process of He-Gel and the tight stacking between molecules, we conducted molecular dynamics simulations. Root Mean Square Deviation (RMSD) is an essential indicator for evaluating the stability of the simulation system [25]. Figure 5A suggested that the RMSD of the entire system mounted rapidly at the beginning with intense fluctuations. After 20 ns, the RMSD tended to stabilize and remained so for a long time, which is a signal that the molecules have aggregated into an integrated whole. It should be noted that in the computational simulations conducted in this study, the assembly process was only simulated for up to 100 ns—a timeframe that is too short to fully capture the dynamics of the assembly process. However, recent studies have demonstrated the feasibility of extending simulation time scales to the microsecond (μs) level [26]. Such an extension would likely reveal that the aggregation of traditional Chinese medicine molecules is not a single-step process, but rather involves multiple distinct stages. This would significantly enhance our ability to elucidate the assembly mechanism. We further calculated the number of intermolecular hydrogen bonds during the simulation process. As depicted in Figure 5B, the average number of hydrogen bonds is 40. This finding emphasizes the crucial role of hydrogen bonds in the He-Gel assembly process. In addition, in Figure 6C, the dynamic co-assembly process of α-hederin molecules is plotted within the framework of aggregate systems at every 20 ns. The final results show that all molecules can aggregate into a lamellar structure, which is consistent with the observations from SEM and TEM.

### 2.5. Low Toxicity and Better Anti-Inflammatory Activity In Vitro of He-Gel

Effective release is an important basis for He-Gel to exert its pharmaceutical effects. Therefore, we investigated the sustained release behavior of He-Gel. Figure 6A shows the in vitro release profile of He-Gel that exhibits a rapid release process of α-hederin in the first 12 h, followed by a slow release process. Subsequently, we fitted its release profile, and the fitting results indicated that the release process of He-Gel conforms to the first-order kinetic equation. This situation indicates that He-Gel possesses a good sustained-release effect.

*Q_t_* = (95.38 ± 1.09) × [1 − *e*^−(0.198±0.008)×*t*^], R^2^ = 0.99934

To assess cytotoxicity, we evaluated the viability of RAW264.7 cells treated with α-hederin and He-Gel for 24 and 48 h using a CCK-8 assay kit. As shown in Figure 6A, both α-hederin and He-Gel exhibited minimal cytotoxicity at concentrations up to 30 μM within the tested range of 3–90 μM. However, after 48 h of incubation, α-hederin significantly reduced cell viability by 44.7% at 30 μM, demonstrating marked toxicity compared to He-Gel (Figure 6B). These results suggest the assembly is conducive to reducing the cytotoxicity of α-hederin. However, it is important to note that both candidates elicited substantial cell death at 60 μM. This dose-dependent toxicity suggests a relatively narrow therapeutic window, indicating that precise dosage optimization is imperative to balance therapeutic efficacy against potential cellular damage in future applications. Subsequently, an inflammatory model was established using LPS-induced RAW264.7 cells, and different concentrations of He-Gel and α-hederin were applied to evaluate their anti-inflammatory effects. After LPS stimulation, RAW264.7 cells undergo marked morphological changes and rapid proliferation, leading to excessive secretion of pro-inflammatory factors and suppression of anti-inflammatory factors. Thus, we detected the concentrations of inflammatory factors in the cell supernatant after 24 h LPS stimulation utilizing enzyme linked immunosorbent assay (ELISA). The results illustrated that the levels of pro-inflammatory factors IL-1β and IL-6 ascended significantly after modeling, which were alleviated after drug administration. Notably, He-Gel exhibited a markedly stronger inhibitory effect than α-hederin (*p* < 0.01 and *p* < 0.05) (Figure 6C,D). IL-10, an anti-inflammatory cytokine that suppresses immune responses and prevents further tissue damage, showed an opposite trend compared with IL-1β and IL-6. As demonstrated in Figure 6E, IL-10 levels increased significantly after treatment, with a more pronounced elevation observed in the He-Gel group compared with α-hederin (*p* < 0.01). These findings suggest that self-assembly not only reduces toxicity but also enhances the therapeutic efficacy of α-hederin. We further detected the expression levels of inflammatory factor mRNAs using quantitative PCR (qPCR). The results showed that after LPS stimulation, the expression levels of pro-inflammatory factors IL-1β and IL-6 increased, while the expression level of anti-inflammatory factor IL-10 decreased. After drug administration, however, the expression levels of IL-1β and IL-6 decreased significantly, and the expression level of IL-10 increased (Figure 6G–I). More importantly, compared with the free form, He-Gel exhibited a superior anti-inflammatory effect. These results are consistent with those of ELISA, which confirms the advantages of the assembly. It is worth noting that while the gel relies on the carboxylate form for assembly, upon dilution in physiological buffers (pH 7.4) for bioassays, both the gel-released species and the neutral control compound re-equilibrate to the same ionization state. Thus, the observed enhancements in bioactivity are attributed to the improved solubility and the supramolecular delivery features of the gel, rather than intrinsic pharmacological differences between the salt and acid forms. In short, the gel exhibited reduced cytotoxicity, enhanced anti-inflammatory activity, and sustained-release behavior. Although we did not conduct in-depth in vivo studies in the present work, we can envision that, when the gel is injected in situ into a tumor, used as a wound dressing, or employed as a carrier for delivering other active molecules, it may serve as a simple-to-formulate, biodegradable, and controlled-release matrix. The gel itself possesses inherent anti-inflammatory activity, ensures safety, and enables the continuous release of active agents, which is beneficial for the development of future novel delivery systems.

Previous studies have shown that α-hederin has a natural affinity for cholesterol in the cell membrane, thereby increasing membrane permeability [27,28]. During this interaction, the hydrophilic sugar moiety is oriented toward the extracellular side, while the triterpenoid moiety embeds into the hydrophobic core of the lipid bilayer. However, the carboxyl group within the triterpenoid portion of α-hederin may generate repulsive interactions with the hydrophobic segment of cholesterol. This repulsion not only promotes the dissociation of α-hederin from the membrane but also contributes to its cytotoxicity. In our study, hydrogen bonds formed by carboxyl groups serve as one of the main driving forces for hydrogel assembly. Therefore, it is likely that the carboxyl groups are not exposed in the hydrogel, resulting in lower toxicity of the assembly compared to the free form of α-hederin. Meanwhile, observation of its microstructure revealed a lamellar structure in the hydrogel, which may increase the contact area between the hydrogel and cells. Additionally, molecular dynamics (MD) simulations demonstrated an increased number of hydrogen bonds in the hydrogel, both of which facilitate better cellular uptake of the hydrogel. Finally, assembled hydrogels typically possess a sustained-release function, which contributes to their continuous release and improved therapeutic efficacy [14].

## 3. Conclusions

In this study, we demonstrated for the first time that the pentacyclic triterpenoid saponin α-hederin can directly self-assemble into a hydrogel (He-Gel) through hydrogen bonding, π–π stacking and van der Waals interactions, without the need for structural modification. The mechanical properties and viscoelasticity of He-Gel were evaluated using rheological and microrheological techniques. To elucidate its self-assembly mechanism, we employed a combination of spectroscopic analyses, further supported by density functional theory (DFT) calculations and molecular dynamics (MD) simulations. In terms of biological activity, we found that He-Gel exhibited relatively lower toxicity and superior anti-inflammatory activity compared with α-hederin. We propose that these advantages are primarily attributable to several factors: (i) in the gel state, carboxyl groups participate in hydrogen bonding, reducing repulsive interactions with the cell membrane; (ii) the microscale lamellar architecture of the hydrogel increases the contact area between the assemblies and cells; and (iii) MD simulations confirmed a higher number of hydrogen bonds in the gel compared to the free form. Nonetheless, this hypothesis requires further validation through additional experiments. In conclusion, compared to the free form, He-Gel shows improved solubility, enhanced therapeutic efficacy, and reduced side effects. This study provides a new insight for the development of α-hederin as a novel anti-inflammatory drug.

## 4. Materials and Methods

### 4.1. Reagents, Cell Line, and Materials

α-Hederin was purchased from PUSH Bio-technology. (Chengdu, China). Sodium carbonate was provided by Beijing Chemical Works (Beijing, China). The water used in this study was ultrapure water, and the remaining solvents were obtained from Beijing Chemical Works. Mouse monocyte-macrophage RAW264.7 cells were purchased from the Cell Resource Center, Peking Union Medical College (PCRC) (Beijing, China).

### 4.2. Preparation of the He-Gel

To form the gel, α-hederin was dissolved in a Na_2_CO_3_ solution, and the mixture was heated at 70 °C for approximately 5 min to obtain a transparent solution. After cooling for 3 h, a homogeneous and stable gel was formed. The formation of He-Gel was confirmed using the test tube tilting method and the inverted vial method. The critical gelation concentration (CGC) was determined to be 50 mg/mL through a controlled variable approach [16]. The detailed procedure is as follows: first, 20 mg of α-hederin was added to 1 mL of Na_2_CO_3_ solution at concentrations of 0.05 M, 0.1 M, and 0.2 M. In the 0.05 M Na_2_CO_3_ solution, α-hederin did not fully dissolve, while it dissolved completely in 0.1 M and 0.2 M Na_2_CO_3_. However, after cooling, no gel formation was observed. Subsequently, the concentration of α-hederin was increased in 10 mg increments. At 50 mg in 0.1 M Na_2_CO_3_, a gel formed after cooling for 3 h. Further increasing the amount of α-hederin resulted in reduced solubility in the 0.1 M Na_2_CO_3_ solution, manifested as a required extension of the heating time. Thus, the optimal conditions for He-Gel formation were determined to be 0.1 M Na_2_CO_3_ and 66.6 mM α-hederin. Finally, the dried He-Gel was prepared using a freeze-drying method.

### 4.3. Morphological Characterization

SEM imaging of freeze-dried He-Gel and α-hederin monomer were investigated at 10 mA, 20 kV (JSM-6700F, JEOL, Tokyo, Japan). TEM image was obtained via a FEI Talos F200X instrument (Thermo, Waltham, MA, USA).

For SEM sample preparation, the gel was first freeze-dried, and a small piece of the dried gel was carefully mounted onto a sample holder with conductive tape, ensuring that the original gel structure remained intact. After gently blowing away any loose powder with an air blower, the sample was coated with gold and then observed. For TEM sample preparation, the dried powders of α-hederin and He-Gel were each dissolved at 1 mg/mL in methanol and water, respectively. A small amount of each solution was then dropped onto a copper grid, and after the solvent evaporated completely, the samples were ready for TEM image.

### 4.4. Micro-Rheological Test

3 mL of He-Sol was prepared in a tailored bottle for optical rheometer test via taking advantage of the method described in Section 4.2. The temperature of the optical rheometer (Rheolaser MASTER™, Toulouse, France) was set at 25 °C. The heated He-Sol was then placed in the optical rheometer to detect its sol–gel transition process. The elasticity index (EI), macroscopic viscosity index (MVI), and fluidity index (FI) were recorded in real time [17].

### 4.5. Rheological Test

First, He-Gel was prepared according to the method mentioned in 4.2. A rotating rheometer (Kinexus, Bavaria, Germany) was employed for rheological analyses, equipped with a 25 mm diameter rotor with an operating clearance height of 1.0 mm and a controlled temperature of 37 °C. Amplitude scanning was recorded from 0.1–100% strain at a frequency of 1 Hz. Single-frequency time sweep was detected at 1% strain, 1 Hz frequency. A shear-thinning detection ranging from 0.1 to 100 s^−1^ was implemented with frequency at 1 Hz [18].

### 4.6. ^1^H NMR Test

Freeze-dried powder of He-Gel and α-hederin were dissolved in 500 µL DMSO-d6 and the signal of proton peaks were obtained at 25 °C via utilizing Bruker AV 700 NMR spectrometer (Bruker, Billerica, MA, USA).

### 4.7. FT-IR Test

FT-IR analysis was conducted using a Thermo Scientific Nicolet IS10 (Thermo, Waltham, MA, USA). Samples were homogenized with spectroscopic-grade KBr, pressed into pellets, and scanned from 4000 to 400 cm^−1^ at 25 °C to obtain the absorption data.

### 4.8. XRD Test

X-ray diffraction analysis was performed on freeze-dried gel powder and α-hederin samples using an X’Pert PRO MPD diffractometer (Almelo, Holland, MI, USA). The powdered specimens, prepared by grinding, were scanned from 5° to 60° (2θ) at a speed of 5°/min.

### 4.9. DFT Calculation

DFT-based calculations and IGMH analysis were performed using Gaussian 16 software, while the compound structures were obtained from the PubChem database.

### 4.10. MD Stimulation of He-Gel

We investigated the molecular dynamics simulation (MDS) of He-Gel in Na_2_CO_3_ solution utilizing the Gromacs 2020.06 software package. The simulation system was constructed by randomly placing 20 α-hederin molecules into a 5 × 5 × 5 nm^3^ cubic box containing an adequate volume of water molecules, using PACKMOL 20.3.5. Prior to the molecular dynamics (MD) simulation, a V-type variable-scale thermostat and the Parrinello-Rahman barostat were utilized to maintain temperature and pressure equilibrium. To remove any potential spatial strain, the system underwent energy minimization via the steepest descent algorithm. During the entire simulation, hydrogen bonds were constrained using the LINCS algorithm, while long-range electrostatic interactions were managed through the Particle Mesh Ewald method. The system described above was simulated over a 100 ns period, allowing for the prediction of the model molecular complex’s RMSD, hydrogen bond count, and simulation trajectories. Conformations were recorded at 20 ns intervals to generate simulation trajectories, and the results were visualized using built-in Gromacs programs and VMD 4.9. The Biomedical High Performance Computing Platform, Chinese Academy of Medical Sciences, supported DFT calculations and molecular dynamics simulations.

### 4.11. In Vitro Release Determination

The in vitro release profile of He-Gel was assessed utilizing the dialysis bag method. Precisely, 1 mL of He-Gel was introduced into a dialysis bag (Spectrum Labs, Gardena, CA, USA; M_w_: 3500 Da) and thereafter incubated in 100 mL of PBS buffer (pH = 7.4, 0.01 M) at 37 °C. At each designated time interval, 1 mL of the sample was extracted for analysis via High-Performance Liquid Chromatography (HPLC, Agilent, Santa Clara, CA, USA), and an equivalent volume (1 mL) of fresh PBS was promptly replaced. The experiment was conducted thrice.

### 4.12. RAW264.7 Cells Culture

RAW264.7 cells grow and passage in complete Dulbecco’s modified Eagle’s medium (DMEM) containing 10% serum and 1% penicillin-streptomycin double antibody, under the conditions of 37 °C and 5% CO_2_.

### 4.13. Cell Viability Assay

RAW264.7 cells were seeded into a 96-well plate at a density of 1 × 10^5^ mL^−1^ in 100 μL of medium and cultured overnight to allow cell attachment. Following stabilization, cells were treated with varying concentrations of the drug for 24 and 48 h, respectively. Subsequently, the culture medium was aspirated, and 10% CCK-8 (Topscience, Wuhan, China) solution was added to each well. After an additional 40-min incubation period, absorbance at 450 nm was measured using a microplate reader (Thermo, Waltham, MA, USA). Here, both the cytotoxicity evaluation and the subsequent assessment of anti-inflammatory activity were conducted by redispersing the lyophilized gel powder in the culture medium for administration.

### 4.14. Elisa Detecting of RAW264.7 Cells’ Supernatants

The anti-inflammatory activities of He-Gel and α-hederin were determined using an ELISA assay. Cells (1 × 10^5^ cells per well in a 12-well plate) were pre-treated with various concentrations of drugs for 2 h, followed by stimulation with LPS (1 μg/mL) to induce inflammation model. After 24 h of treatment, cell supernatants were collected. The concentrations of IL-1β, IL-6, and IL-10 in the culture medium were measured using ELISA kits according to the manufacturer’s instructions (Elabscience, Wuhan, China).

### 4.15. qPCR Determination

According to previous study [29], total mRNA was extracted from RAW264.7 cells subjected to different treatments, and the expression levels of inflammatory cytokines in these differently treated RAW264.7 cells were detected using quantitative polymerase chain reaction (qPCR).

## Data Availability

The original contributions presented in this study are included in the article. Further inquiries can be directed to the corresponding authors.
